# Evaluation of the Behaviour of Steel Bar in the Concrete under Cyclic Loading Using Magnetic Flux Leakage and Acoustic Emission Techniques

**DOI:** 10.3390/ma16062172

**Published:** 2023-03-08

**Authors:** Noorsuhada Md Nor, Shahrum Abdullah, Mohamad Afiq Hazwan Mohamad Halim, Azli Arifin

**Affiliations:** 1 Civil Engineering Studies, College of Engineering, Universiti Teknologi MARA, Cawangan Pulau Pinang, Kampus Permatang Pauh, Permatang Pauh 13500, Malaysia; 2 Department of Mechanical and Manufacturing Engineering, Faculty of Engineering & Built Environment, Universiti Kebangsaan Malaysia, Bangi 43600, Malaysia; 3 Astana Setia Sdn. Bhd., Wisma Lim Seong Hai, 33 Jalan Gombak, Wilayah Persekutuan, Kuala Lumpur 53000, Malaysia

**Keywords:** acoustic emission, magnetic flux leakage, cyclic loading, failure analysis

## Abstract

The behaviour of the steel bar in concrete under cyclic loading has been evaluated using magnetic flux leakage associated with acoustic emission monitoring technique. Visual observation was used to observe the deformation of the beam under cyclic loading. The sensors of metal magnetic memory were scanned in the middle of the beam at a distance of 320 mm at the bottom part. Twenty-two cyclic ranges were performed for cyclic loading of 100 or 200 cycles for each range, with a frequency of 1 Hz and a sinusoidal wave mode. The magnetic flux leakage signal, acoustic emission characteristics and crack width were measured and analysed to evaluate the behaviour of the steel bar in the concrete beam. The magnetic flux leakage signal and acoustic emission energy results were well matched with the occurrence of cracks at the centre of the beam. It was found that the relationship between the magnetic leakage flux signal and crack opening showed a strong correlation with R^2^ of 0.969. A high acoustic emission energy of 1300 nVs is observed at the centre of the beam. Based on the results, the behaviour of the steel in the concrete beam can be determined by the integrity assessment of a structure.

## 1. Introduction

The behaviour and integrity of the steel embedded in concrete plays a crucial role in ensuring that the two materials can work together. The combination of these two materials is the most widely used in construction worldwide, as it has excellent mechanical performance due to the high compressive strength of the concrete and the excellent tensile strength of the reinforcing steel [1,2,3]. As reinforced concrete (RC) constructions are made up of brittle concrete and ductile steel, residual micro-crack coalescence of concrete may emerge because of increased steel strain, which reduces the adhesion of these two materials. Hence, the reliable behaviour of steel bars in concrete can lead to good serviceability of reinforced concrete structures [4,5]. In addition, the steel bar embedded in the concrete is used to hold the concrete together, which can prevent the occurrence of large cracks and increase the overall strength of the reinforced concrete structure, especially when the structure under cyclic loading.

As RC structure is a popular material in construction, e.g., for building bridges and carriageways for high-speed railways and dams, they are subjected to cyclic loads during their service life. This type of loading affects the performance and integrity of the structures as the performance decreases [6]. The effects not only affect the concrete, but also the steel bars embedded in the concrete, as the load acts continuously. Noorsuhada [7] has provided an overview of the effects of cyclic loading on reinforced concrete structures. It was found that the loading leads to the formation of cracks in the concrete until failure, and requires monitoring of the structures during operation so that the integrity of the structure can be monitored. Based on the results of the study, a monitoring technique using a non-destructive technique (NDT) as well as acoustic emissions is strongly recommended. Saliba and Mezhoud [8] investigated the steel–concrete bond with acoustic emission technique under pull-out. They found good correlation between loading force and acoustic emission activity as the fracture mechanism activity differed. This is because the acoustic emission activity is highly correlated to the different degradation states of the bonding between steel and concrete. In addition, acoustic emission can provide additional insight to the behaviour of reinforced concrete structures. Tsangouri and Aggelis [9] claimed that the effectiveness of reinforcement in concrete and other cementitious composites can be indicated by acoustic emission. Chai et al. [10] stated that acoustic emission data can be used to identify and predict the growth of fatigue cracks at different stress ratios. Acoustic emission also provides some advantageous such as early crack monitoring, the ability to locate damage and prediction of future failure [7]. Although it is advantageous, some limitations were also noted, as the integrity of the steel in the concrete cannot be directly detected by the acoustic emission technique. This is because the accuracy of the localisation of the acoustic emission characteristics depends on several parameters such as the sensor layout, the accuracy of the arrival time picking, the velocity model, and the propagation path of the wave [8]. Moreover, when the steel and concrete have different wave velocities and reflections at interfaces and heterogeneities, the localization result can be significantly influenced [11]. Therefore, other tools are recommended to investigate the behaviour of the steel in the concrete.

Most studies on the behaviour of steel in concrete are based on the bond between these two materials under pull-out [8,12,13,14]. Normally, the bond stress and the relationship between bond stress and slip between these two materials have been determined. However, the behaviour of steel bars embedded in concrete under cyclic bending load has not been sufficiently investigated. Since other non-destructive tests are required to study the behaviour of steel in concrete, a respective device such as the metal magnet memory (MMM) method can be used for this purpose. Sahadan et al. [15] investigated whether the MMM method can be used to assess ferromagnetic materials under fatigue damage caused by changes in density and lattice dislocation patterns, as well as the generation of continuous slip bands that cause changes in magnetic properties or magnetic flux leakage.

Magnetic flux leakage (MFL) is a passive NDT technique based on magneto-mechanical effects. MFL has been used extensively to detect corrosion on steel strands [16], to evaluate fatigue damage on tower crane pulleys [17], to inspect pipeline systems [18], and to detect losses in the metallic domain in external prestressing systems [19]. Firdaus et al. [17] found that MFL is useful to detect the location of the defect on the damage tower crane pulley. It can also be used to detect the failure mechanism of ferromagnetic materials at an early stage. Karthik et al. [19] emphasised that the MFL is valuable in detecting, locating and estimating loss in the metallic area of an external post-tensioning girder during the bridge in service. The estimation of the failure mechanism is presented from the component signals (normal and tangential component signals) collected during scanning by sensors of the ferromagnetic materials on the surface.

Although the MFL technique is extensively used for detection damage of ferromagnetic materials, the use of this technique on detection fatigue damage of reinforced concrete structures is now starting to be reported, for example, by Xie et al., who fixed the MMM in specific locations [20]. They inferred that the MMM signal is useful for characterising the fatigue damage process of the composite specimen because the magnetic flux intensity changes rapidly in the first stage, develops steadily and slowly in the second stage, and distorts in the third stage, which corresponds to the three-stage fatigue damage development trend. Gong et al. [21] have used MMM to measure stress in the RC beam under four-point bending. They found that the quantative measurement of the working stress of the reinforced concrete structures can be preliminarily achieved. Karthik et al. [19] have investigated the application of MMM in the metallic area in the control girder of the external post-tensioning systems of bridge. They found that the magnetic flux leakage can be used to detect the loss in metallic area, although some errors were found, but however, it gives a good indication on the severity of the defect on strands in a tendon. This indicates that the MFL technique is relevant for use in measuring the behaviour of steel bars embedded in the concrete, as investigation of this scenario is currently still limited. Although the MMM is beginning to garner attention for measuring steel bars in concrete, the relationship between magnetic flux leakage signal and acoustic emission is also still limited.

The aim of this study is to experimentally investigate the behaviour of steel bars embedded in concrete under cyclic loading, using the MMM and verified by acoustic emission technique. It was taken into consideration that the embedded steel bar was 20 mm from the bottom section or the tensile part of the beam. The MMM sensor was used to scan the soffit of the beam when the cyclic load stopped for specific load cycles. Meanwhile, the acoustic emission was used to monitor any displacement that occurred during testing. Then, equations between the magnetic flux leakage, acoustic emission characteristics and the crack opening were proposed. This study is significantly beneficial for predicting the behaviour of the steel bar in the concrete during service and for helping to maintain the integrity of the RC beam. Early detection of the condition of the concrete and the steel in the concrete can help to maintain the performance of the structure as a whole, especially for infrastructures such as bridges, which are subjected to cyclic loads as vehicles cross the bridge. Since the strength of the structures is highly dependent on the strength of the steel, the magnetic flux leakage associated with the acoustic emission technique is a useful tool to determine the flaw of the steel at an early stage, which eventually may lead to crack and final fracture.

## 2. Materials and Methods

**Figure 1:**
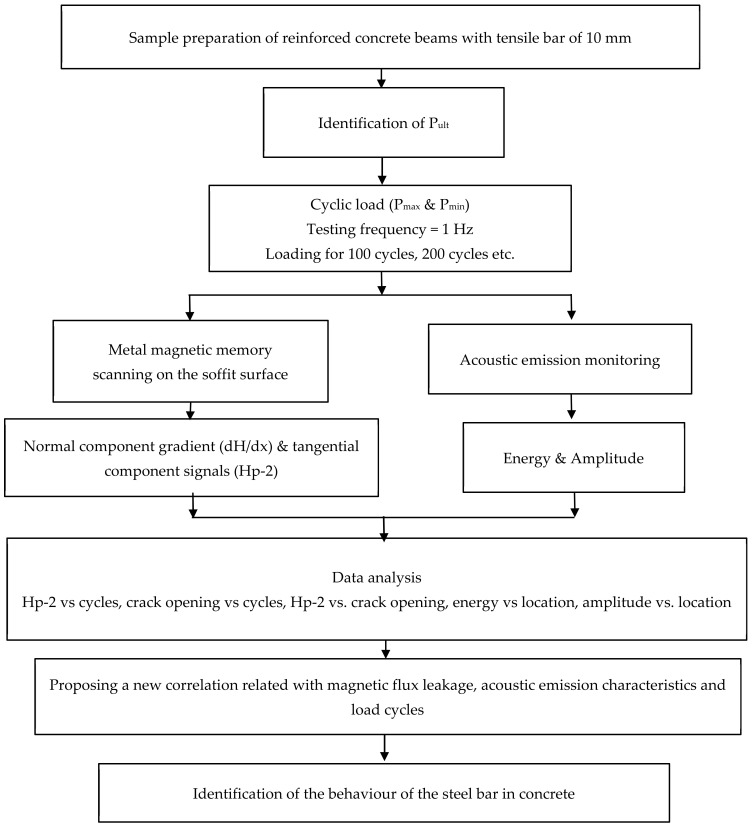
Process flow for evaluation of the steel bar behaviour in concrete beam.

The process flow of evaluation of the behaviour of the steel bar in a concrete beam using the MMM method is presented in Figure 1. It started with the preparation of the sample of concrete beam; a10 mm steel bar was inserted in the beam at a certain position on the tension part. Prior to carrying out the cyclic loading, the static test was identified for determination of the ultimate load of the RC beam, and the value was used for identification of the maximum load, Pmax and minimum load, Pmin, that were applied for the cyclic test. The Pmax that was applied for the cyclic test was 70% of the ultimate load of the beam. The ultimate load of the beam was 22.7 kN. Hence, the Pmax used for this study was 16 kN. The Pmin was set as 5 kN as the minimum load of the machine can be applied for the cyclic loading. The cyclic test was carried out with constant frequency of 1 Hz and ran for 100 and 200 cycles. A frequency of 1 Hz was applied in order to investigate the acoustic emission activities during low cyclic loading. The 100 cycles or 200 cycles were applied to investigate the behaviour of the beam at the short cycles. The loading was applied again based on the loading protocol applied for this test. The MMM method was used to measure the magnetic flux leakage by scanning at the specific surface of the beam on the soffit. The normal component gradient, dH/dx and tangential gradient signal Hp-2 were then identified. Meanwhile, from the acoustic emission monitoring, the energy and the amplitude were determined. The magnetic flux signal was then identified and analysed. The correlation between several parameters was identified with the indication of good correlation (R^2^ > 0.8) [22] between respective parameters. If good correlation was achieved, a new correlation was then identified. The behaviour of the steel bar in concrete was then identified.

### 2.1. Preparation of Samples

A total of six reinforced concrete beams were prepared. The high strength steel bar of 10 mm (T10) diameter was used in this study. Table 1 presents the mechanical properties of the steel bar, which was tested and reported in the previous work [23]. The concrete was designed in accordance with standard BS 8110 [24] using grade C30 which contained cement, fine aggregate and coarse aggregate of 1, 1.53 and 2.12, respectively. A constant water/cement ratio of 0.5 and superplasticizer of 0.5% of cement weight was used for the concrete mix, to boost the workability of the fresh concrete.

**Figure 2:**
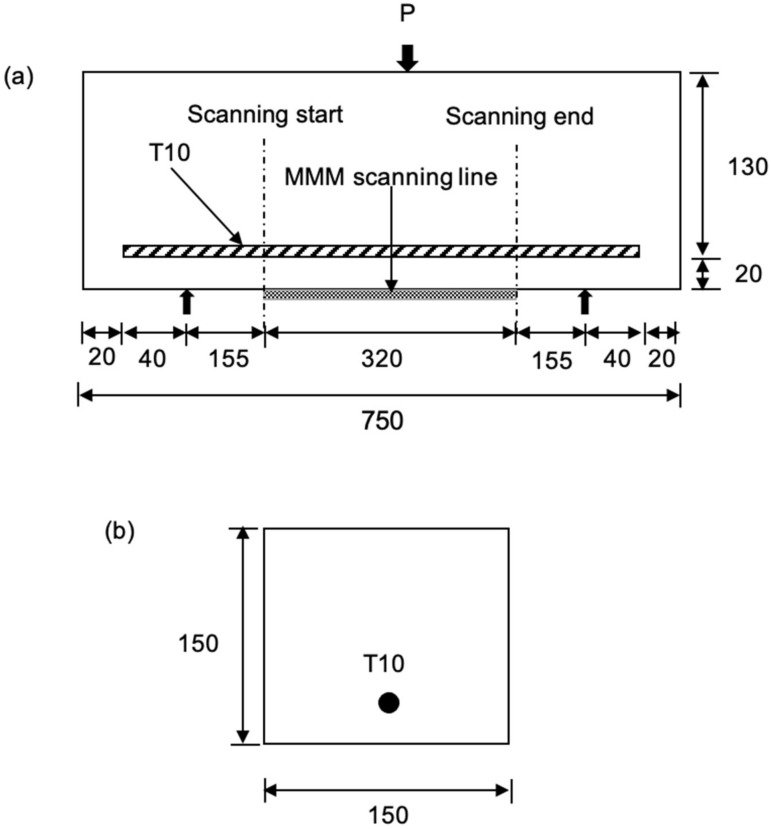
Schematic diagram of the RC prism details (**a**) side view (**b**) cross-sectional view (unit in mm).

**Figure 3:**
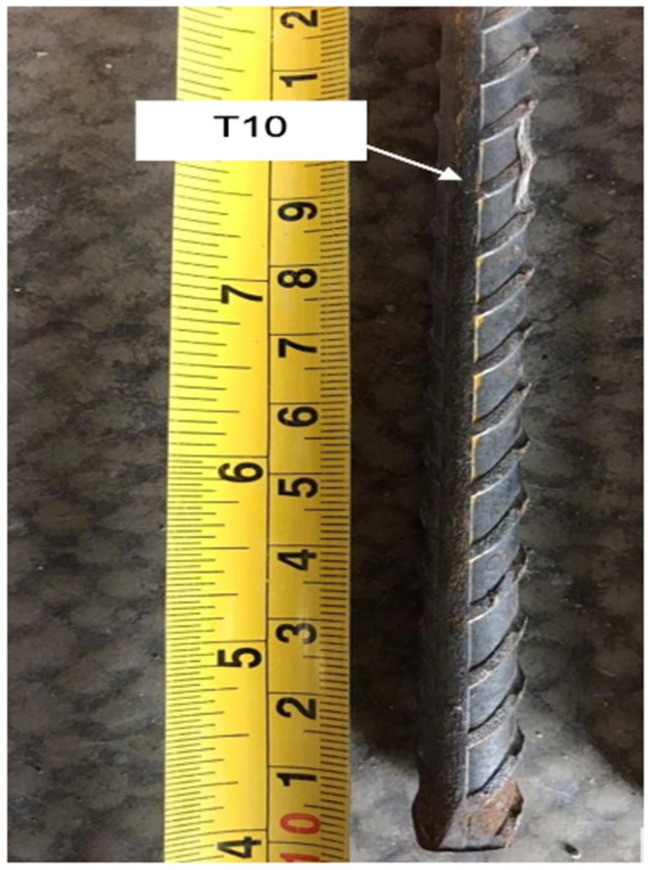
The high yield steel’s T10.

Figure 2 shows the schematic diagram of the 750 mm length RC beam; a steel bar (see Figure 3) (length 710 mm) with a cover of 20 mm has been inserted in the concrete and is located in the middle of the beam profile. To ensure that the steel bar is in the centre of the beam and does not slip when the concrete is poured, spacers were used to hold the steel bar.

### 2.2. Experimental

Two types of tests were performed, i.e., a static test and a cyclic test. The static test with 3-point bending was performed to determine the ultimate load of the beam with a constant loading rate of 1 mm/min according to ASTM C78 [25]. From the static test, it is found that the ultimate load for concrete beam that has diameter of 10 mm was 22.7 kN. For cyclic loading, the loads were based on two boundaries of maximum load, Pmax and minimum load, Pmin. After determining the ultimate load from the static test, the load was used to determine the Pmax for cyclic loading. The value of the Pmax for each specimen for cyclic loading was set at 70% of the value of the ultimate load from the static load to prevent the specimens from failing directly when the maximum load was applied to the surface of the specimens [22]. The Pmax therefore becomes 16 kN. The Pmin was set at 5 kN due to compatibility of the machine. The lowest value with which the machine can work well is 5 kN.

**Figure 4:**
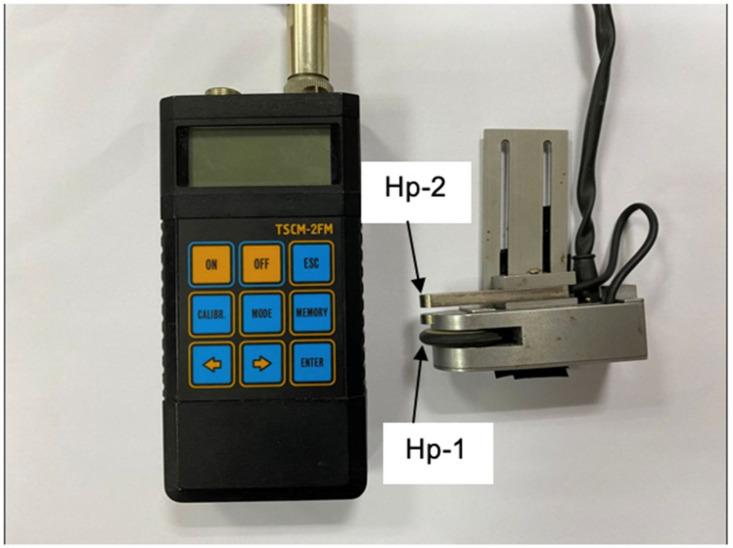
Tester of stress concentration magnetometric.

The cyclic test was based on a constant amplitude loading pattern with 100 or 200 cycles for 2 min at the frequency of 1 Hz until the specimens failed. The acoustic emission was applied to monitor the occurrence of damage that appeared on the beam surface. It was performed in conjunction with cyclic loading for each load range. The machine was stopped every 100 or 200 cycles (see Table 2) to measure and observe cracks. The metal magnetic memory (MMM) was used to capture the magnetic flux leakage signal at the respective location at the two sensors of Hp-1 and Hp-2 (see Figure 4). The description of the Hp-1 and Hp-2 will be discussed in the following sub-section.

The cyclic loading arrangements as set in Table 2 were from 0–100 cycles, up to 1000 cycles with a 100-cycle interval. After 1000 cycles, the load increased, with a 200-cycle interval up to 3000 cycles. At 3000 cycles, more cracks could be observed and the crack widths were widened. This load arrangement was used in order to investigate the crack on the concrete. At the same time, the behaviour of the steel bar in the concrete can be investigated using MMM technique.

### 2.3. Metal Magnetic Memory

The MMM method was used to measure the performance of the steel bar in concrete when the beam is subjected to cyclic loading. This method, based on the measurement of magnetic signals on the surface of the specimen, is a non-destructive test and can be used to track and monitor the fatigue growth failure of the specimen [26]. Using this method, the initial failure of the components of the ferromagnetic material can be characterised by magnetic signals, especially in areas of stress concentration. Two component signals were used to detect damage to ferromagnetic materials, namely normal component signals, HP (y) or Hp-1 and tangential component signals, Hp(x) or Hp-2, as an indication of the area of the stress zone [27]. The height of the stress zone on the sample can be measured by the largest value of the normal component gradient, dHp(y)/dx, and potentially used to track the progression of cracks [28]. This is because magnetic saturation occurs when an external magnetic field of sufficient strength is applied near a ferrous material, causing all the atomic dipoles of the ferrous substance to align with the external magnetic field. The stress zone region is represented by the location at which the magnetic flux “leaks” due to a reduction in the cross-sectional area, defects, and any changes in the ferromagnetic materials. These can be identified by scanning the sensors placed between the magnetic poles to indicate the location of defects in the steel sample [29].

**Figure 5:**
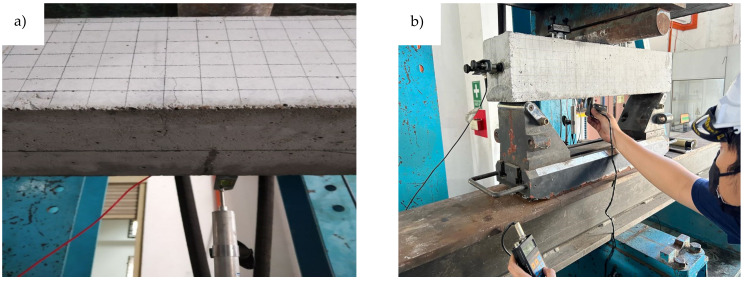
(**a**) The location of scanning pathway (**b**) scanning of the beam after it is subjected to cyclic loading.

The MMM was based on the magnetometric stress concentration test set (TSCM) as shown in Figure 4. It was connected to two parts, i.e., the sensor device for data observation and the computer for analysis. This type of sensor has a wheel and two sensors called Hp-1 and Hp-2. The sensor is used to observe magnetic signal data, and the wheel is an additional sensor for distance determination. The sensing distance was set to 320 mm to obtain suitable signal results, and the distance mode was used to detect the magnetic signal data, as presented in Figure 5. This mode is suitable for detecting and monitoring stress concentrations at the crack site [26]. In this mode, the sensor device is mobilised at a certain distance based on the sensor wheel on the rail.

In this study, the magnetic signal is observed at no load, from the beginning of the test, during the ongoing test and until the samples fail. The scanning at no load was carried out to observe the influence of the environmental magnetic field on the results [29]. The position of the sensor device is perpendicular to the surface of the specimen where the crack propagates on the surface of the specimen. In this position, the device can observe magnetic signal data at the point of stress convergence [30].

### 2.4. Acoustic Emission Monitoring

**Figure 6:**
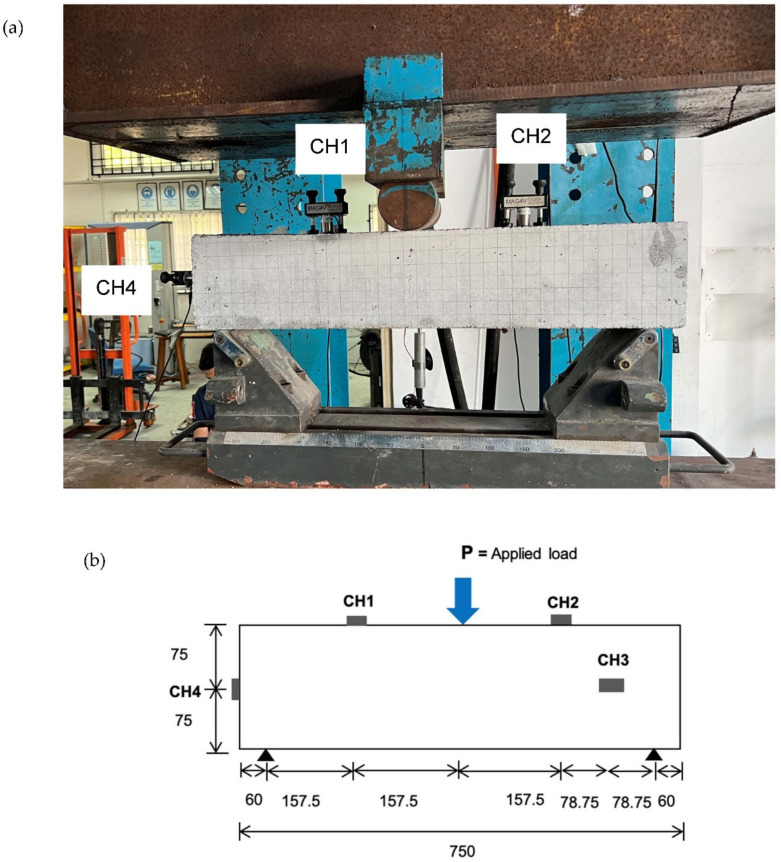
(**a**) Location of the acoustic emission sensor on the beam surface (one sensor, CH3 was located at back side of the beam) (**b**) Schematic diagram of the test set-up and all sensors.

For the acoustic emission monitoring, four sensors, type VS75-V, which have a peak frequency of 75 kHz and pass-band of 30–120 kHz, were coupled on the beam at a specific location. The high vacuum grease was used as a coupling between the sensor surface and the beam surface. Two sensors were coupled on top of the beam, designated as CH1 and CH2 and located at 217.5 mm and 532.5 mm from the edge of the beam, as shown in Figure 6a. One sensor was coupled at centre of the beam on its cross section, and another sensor at 138.75 mm. Figure 6b shows the schematic diagram of all sensors fixed on the beam surface. All sensors were firstly calibrated using a pencil lead fracture (PLF) in order to keep good contact between the sensor and the beam surfaces. Prior to performing the acoustic emission monitoring, the threshold level was set at 35 dB with a wave velocity of 4000 m/s. In the acoustic emission hardware, the parameters were set at a rearm time of 1.62 ms, a sampling rate of 10 MHz, a duration discrimination time of 400 µs and pre-trigger samples of 200. The digital setting was set from 25 kHz to 850 kHz. The results were digitised, stored and visualised. For each cycle range, the crack opening at the middle of the beam was measured and recorded.

### 2.5. Data Analysis

The crack width on the beam surface was measured with the digital caliper when the machine stopped. The crack propagation was then recorded, and a torch was used to observe the appearance of the hairline crack on the beam surface. Meanwhile, the signal of magnetic flux leakage obtained by the MMM method was analysed to determine the area of stress concentration and the location of crack initiation and its growth. The characterisation of the magnetic signals is the gradient signal of the normal component, dh(y)/dx with a fatigue growth parameter in a stable crack growth region. The dh(y)/dx signal is used in crack growth analysis compared to the H(y) signal because the dh(y)/dx signal is more sensitive to the degree of damage to the sample [17]. However, in this study, it is found that the dh(y)/dx signal can be used to interpret the crack growth behaviour at different stress ratios. The magnetic signal obtained by the magnetic memory method is released in the region of stress convergence in the area in which the cracks propagate. This magnetic signal also illustrates the degree of damage to the sample [28]. The acoustic emission energy has been collected based on the location of four sensors that set as a planar plane to capture the waveform. The trend of scattered energy and amplitude were then analysed to identify the critical location of the beam when subjected to cyclic loading.

## 3. Results and Discussion

### 3.1. Magnetic Flux Leakage Signal Analysis

**Figure 7:**
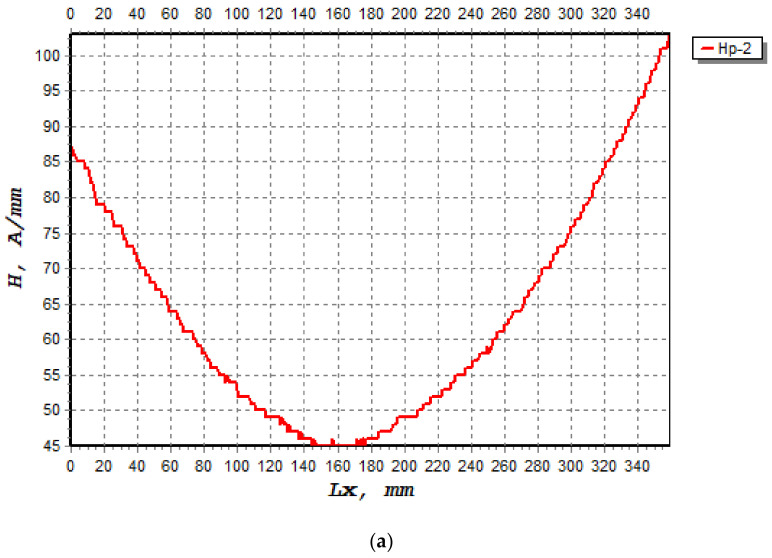

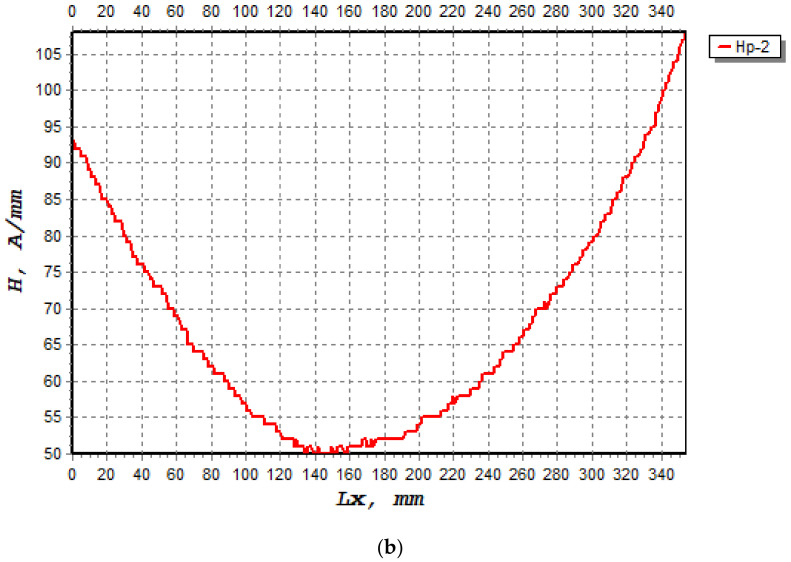
The component signals, H(p), that occurred on the beam when subjected to cyclic loading; Hp-2 during load cycles (**a**) 1400, (**b**) 2800.

**Figure 8:**
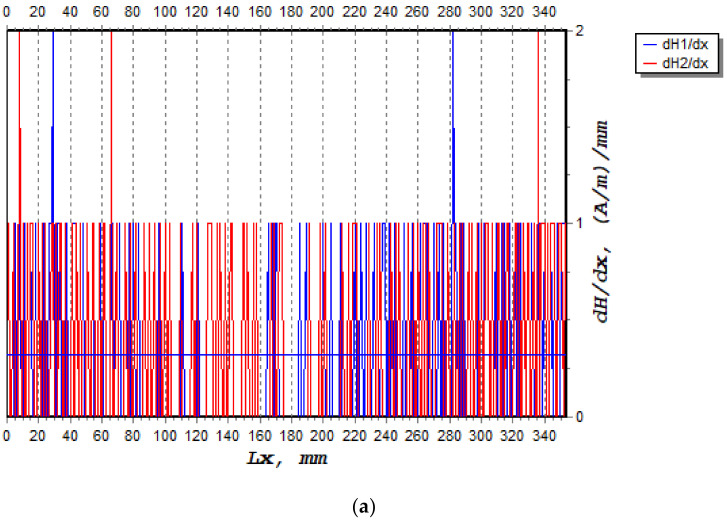

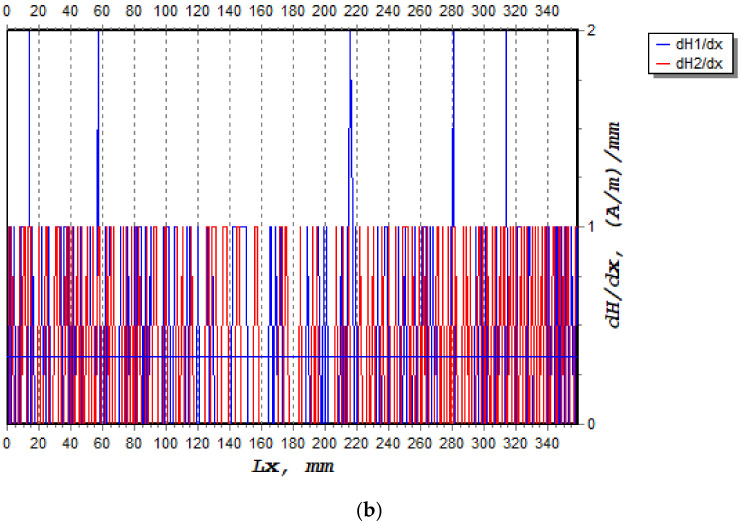
The normal compound gradient signals, dH(y)/dx that occurred on the beam when subjected to cyclic loading: (**a**) 1400 cycles, (**b**) 2800 cycles.

The magnetic signal triggered by the ferromagnetic bar undergoing crack growth was recorded. The variation of the magnetic signal value is a function of the concentration area experienced by the beam under cyclic loading. The characterisation between the magnetic signal parameters is based on the crack growth areas at the scan line, as shown in Figure 7 and Figure 8. Two magnetic leakage flux signals were determined, namely component signals, H(p) and normal gradient signals, dH(y)/dx, as shown in Figure 7 and Figure 8, respectively. Only component signals H(p) were presented, as this H(p) signal consists of two response signals of the signals Hp-1 and Hp-2. For this work, only one signal was considered, namely the Hp-2 sensor, which provided acceptable data compared to the Hp-1 sensor. Technically, this Hp-2 signal show high magnetic gradient values, indicating a high stress concentration in this region.

**Figure 9:**
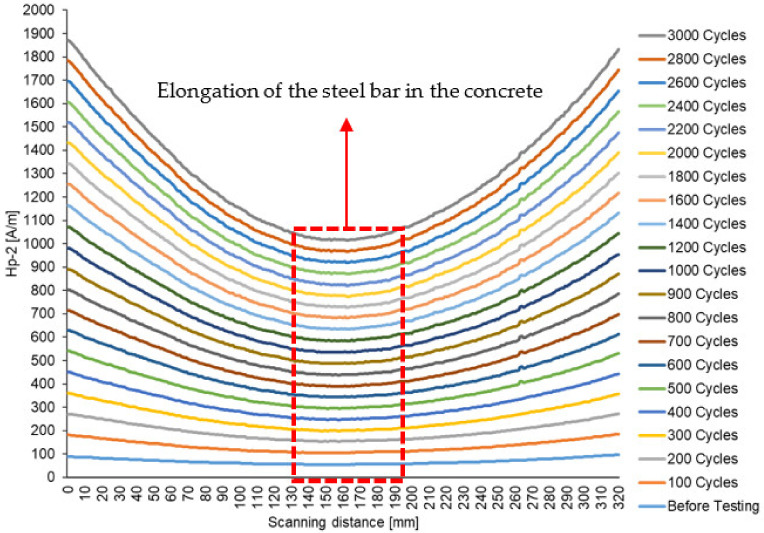
Magnetic flux leakage signals with scanning interval distances showing the stress concentration zone on the beam.

**Figure 10:**
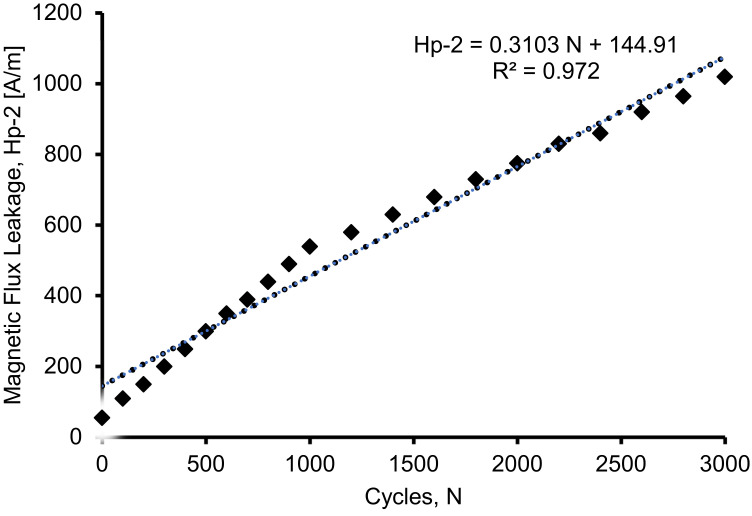
Magnetic flux leakage signals at critical distance for 10 mm high yield steel bar.

Figure 9 shows the graph of the Hp-2 magnetic leakage flux signal (A/mm) against the scan distance of 320 mm before the beam is subjected to cyclic loading to 3000 cycles. It is clear to see that the magnetic leakage flux signals take on a convex shape, indicating that the shape of the magnetic signals changes significantly due to changes of steel bar in the beam during cyclic loading. The changes in the magnetic signal pattern before and after the cyclic loading of the wearer can be clearly seen. For the time before the test, it can be seen that at a distance X (mm) of 0 to 320 mm, no significant changes can be seen in the Hp-2 signals. This is due to the fact that there is no stress concentration, as there is no load on the sample. When the beam is subjected to cyclic loading (see Figure 10), it is found that the shape of the Hp-2 signals changes along the scan distance, with a much smaller signal seen at a distance between 147 mm and 189 mm. In this range, the leakage flux signal is at the lower peak, indicating elongation of the steel bar in the concrete beam. The lower peak of the signal was found at the mid-span of the beam, or at 389 mm from the edge on the left side of the beam.

The changes in the signals show the evolution of the crack in the sample [31]. It can also be seen that the signal pattern in the centre of the scan line takes on a convex shape. By analysing the Hp-2 signal, the location of crack growth can be identified in the areas in which the signal is lowest, indicating that the zone of stress concentration is dissipating. According to Liang et al. [32], different signals are observed in each sample, because it has a different processing history and the sample itself is not homogeneous.

### 3.2. Relationship between Magnetic Flux Leakage and Load Cycles

**Figure 11:**
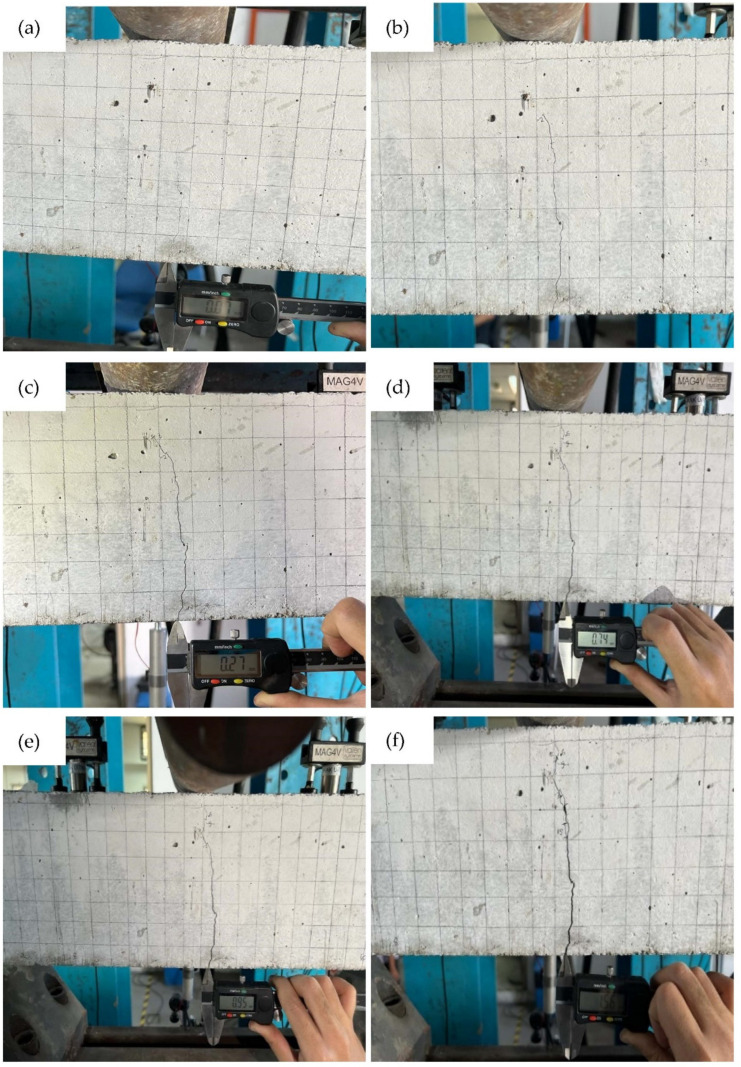

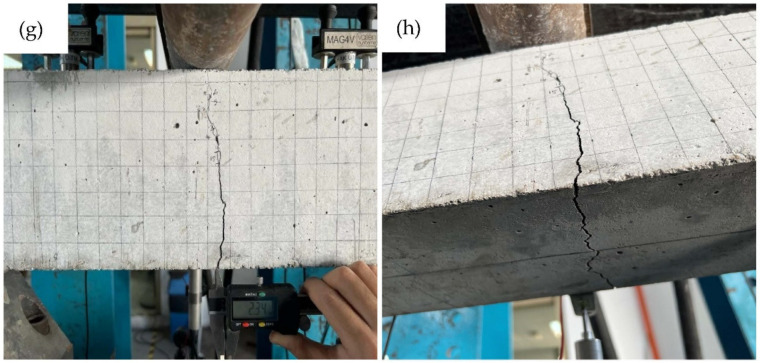
Crack pattern of the beam when subjected to cyclic loading at (**a**) first 100 cycles (**b**) 100 to 200 cycles (**c**) 300 to 400 cycles (**d**) 700 to 800 cycles (**e**) 1000 to 1200 cycles. (**f**) 1800 to 2000 cycles (**g**) 1800 to 2000 cycles (**h**) bottom part of the beam.

Figure 11 shows the correlation between magnetic flux leakage and load cycles for a beam with a steel bar of 10 mm diameter. It is clear from the figure that the magnetic flux leakage signals increase significantly with load cycles. The plotted point was taken at the lowest point of the magnetic leakage flux signals for each cycle wherein the stress concentration zones were detected. From the recorded data of the Hp-2 signals, it was found that the plot pattern increases linearly at each cycle. The result shows a good correlation between the magnetic flux leakage and the load cycles, with a value of R^2^ of 0.9725. When the R^2^ is above 0.9, this indicates a strong correlation between the two variables. The correlation of the magnetic flux leakage and the cycles is presented in Equation (1).

Hp-2 = 0.3103 N + 144.91(1)

where Hp-2 is the component signal captured by sensor Hp-2, and N is the cyclic loading.

Figure 10 shows that before 1000 cycles, there is a linear correlation between the magnetic flux leakage and the load cycles. This indicates that the steel in the concrete beam is beginning to deform. However, the deformation of the steel becomes aggressive after 1000 cycles and shows another linear correlation up to 3000 cycles.

### 3.3. Crack Pattern of the Reinforced Concrete Beam under Cyclic Loading

Figure 11 shows the crack pattern of beam subjected to cyclic loading of 0–100 cycles, 100–200 cycles, 300–400 cycles, 700–800 cycles, 1000–1200 cycles, 1800–2000 cycles and 2800–3000 cycles. Crack width was measured for each cycle range. From the crack patterns, it can be seen that as the cyclic loading increased, the crack propagation increased and the crack width also increased. It was found that the crack displacement during the cycles shows that the crack has started to propagate. Observation with the digital calliper shows that the crack width is less than 1 mm at each cycle when the beam is loaded between 0 and 1200 cycles. Although the beam is subjected to 1200 cycles, a crack width of 0.95 mm was measured. Based on the crack observation, the occurrence of cracks in the concrete started at earlier cycles from 0 to 100 cycles. This was due to the fact that the compressive strength of the concrete was not able to support the minimum and maximum loads of the beam. In this work, the minimum and maximum loads were set at 5 kN and 16 kN, respectively. For example, at 100 load cycles, a hairline crack with a width of 0.07 mm was visually observed (see Figure 11a). At this load cycle, the crack propagated vertically from the lower part to beyond the neutral axis. This indicates that the concrete of the beam started to fail. As noted by Qiu et al. [33], continuous loading would cause the crack to propagate from the top of the previous crack and cause the failure of the specimen.

The continuous cyclic loading increases the crack opening by 0.18 mm at 100 to 200 cyclic loadings (Figure 11b). Crack observation at 300 to 400 cyclic loads shows that the crack grows slightly from the previous crack, with a crack width of 0.27 mm (Figure 11c). Although some cracks did not propagate from the tips of the previous crack, the crack widths were continuously increased; as an example, Figure 11h shows the crack from the bottom part of the beam.

**Figure 12:**
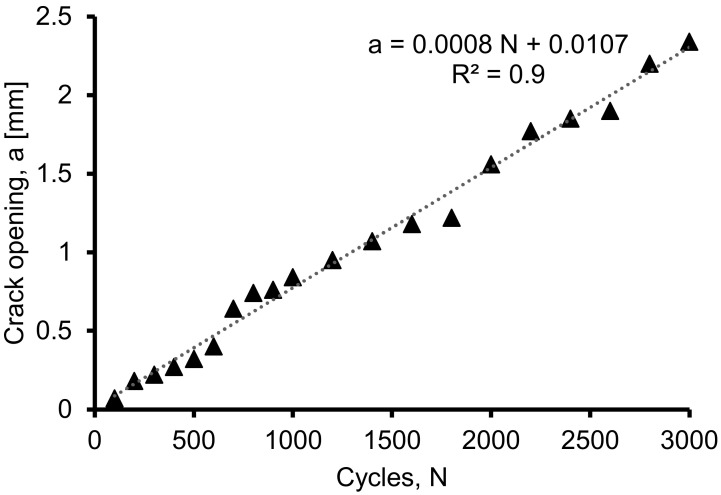
Relationship between crack opening and the load cycles of the beam.

Figure 12 shows the relationship between crack opening and load cycles. Measuring the crack pattern of the beam in relation to the range of load cycles is vital in determining the progression of damage until the failure of the beam. The figure shows a strong correlation, with an R^2^ value of 0.99 between the crack width and the load cycles. The correlation is based on the linear best fit plot, and the equation of this correlation is presented in Equation (2).

a = 0.0008 N + 0.0107(2)

where a is defined as the crack opening (measured in mm) and N is the cyclic loading.

**Figure 13:**
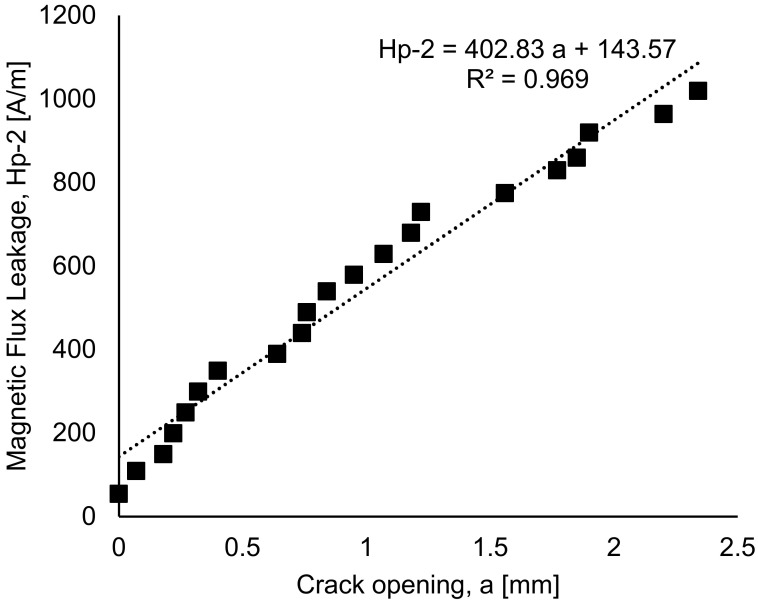
The correlation between magnetic flux leakage and the crack opening.

Figure 13 shows the relationship between the magnetic flux leakage and the crack opening, presenting a strong correlation between the two variables, with an R^2^ of 0.969. Hence, from this correlation, Equation (3), based on the magnetic flux signal and the crack opening, was proposed as follows:

Hp-2 = 402.83 a + 143.57(3)

where Hp-2 is the component signal from scanning of sensor MMM, and a is the crack opening when the beam subjected to cyclic loading.

This correlation is useful for estimating the integrity of the beam, especially as it relates to the cracking and behaviour of the steel bar in the concrete. The prediction of the crack growth from the initiation of the crack in the concrete until failure can be made. The behaviour of the steel bar in the concrete can also be estimated using the equation pronounced by the relationship between magnetic flux leakage signal and crack opening.

### 3.4. Acoustic Emission Characteristics

**Figure 14:**
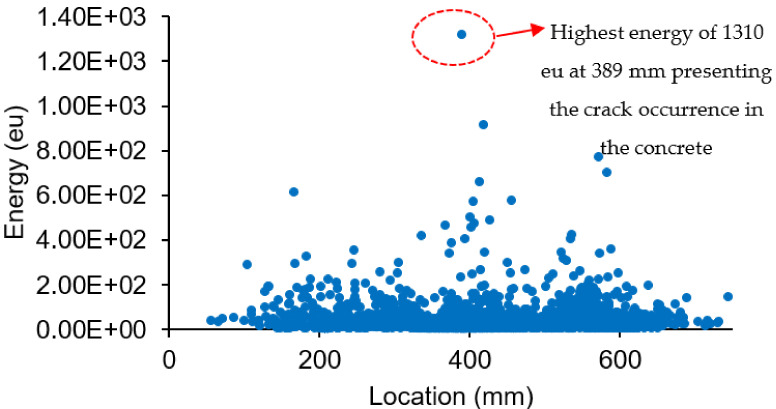
Energy distribution with respect to location.

**Figure 15:**
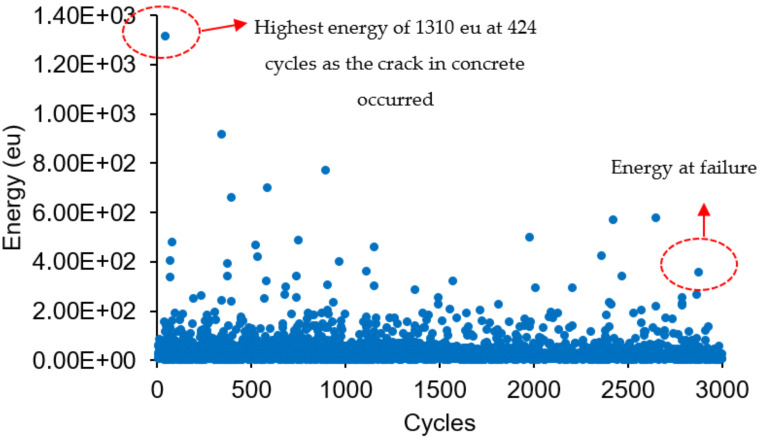
Energy distribution along the cyclic loading.

**Figure 16:**
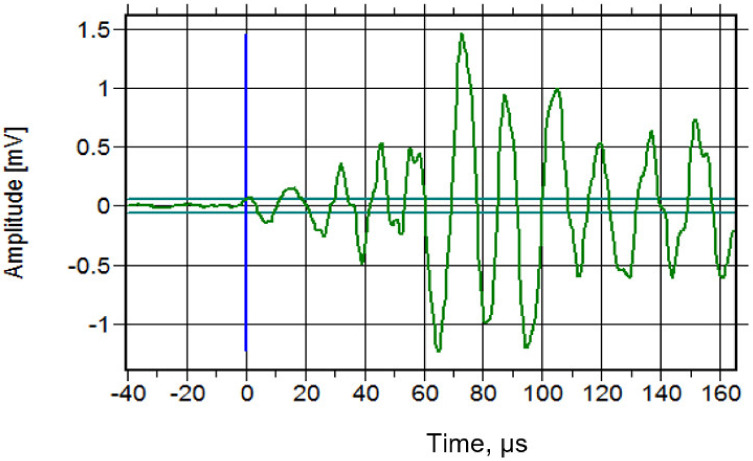
The transient wave at the highest energy.

**Figure 17:**
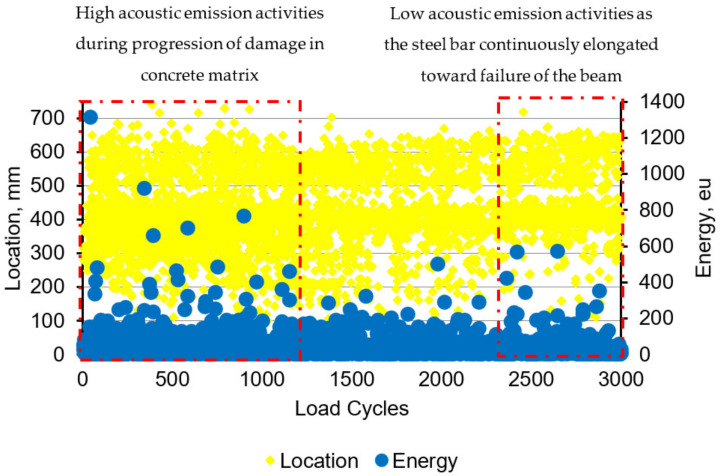
Distribution of the acoustic emission activities based on the location.

Figure 14 shows the distribution of acoustic emission energy along the beam length. High acoustic emission energy is seen at a distance 300 mm to 500 mm from the edge of the left beam. The highest energy was shown at the location 389 mm from the left side of the beam, which is due to the deformation of the beam producing a high stress concentration at this location. The highest acoustic emission energy at this location was 1310 eu. The energy distribution along the load cycles is shown in Figure 15. The highest energy occurred at 424 cycles, with the fifth cycle between 400 and 500 cycles. From this distribution, it can be seen that the highest energy occurs in the first 900 cycles, and the energy decreases as the load increases up to certain load cycles. This high acoustic emission energy creates a transient wave, as shown in Figure 16, with a value of 1.48 mV. From the amplitude of the acoustic emission, a high value can be seen at the point at which the high acoustic emission energy was detected. Figure 17 shows the acoustic emission activities and the distribution of locations along the cyclic load, with high activities being seen until the load reaches 1200 cycles. These high activities are due to the progression of damage in the concrete matrix and the deformation of the steel bars under the continuous cyclic loading.

**Figure 18:**
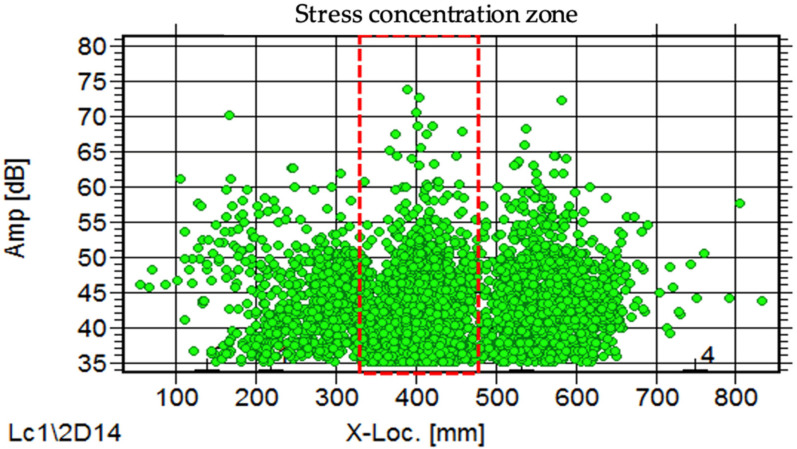
Acoustic emission amplitude along the span of the beam.

The same activities can also be observed by looking at the relationship between amplitude and locations, as can be seen in Figure 18. A high amplitude of 74 dB can be observed at the same location, indicating high stress concentration at that location. Similar results have been found in Li et al. [34], where there is a sudden increase in the activity of AE as stress increases, and the amplitude of the signals continues to rise unexpectedly. The location of this highest acoustic emission energy and amplitude was where the low magnetic flux leakage signal from Hp-2 was detected when scanning the MMM. Since the MMM can be used to detect changes in ferromagnetic materials, these two methods of acoustic emission and MMM are useful for detecting the location of a weak steel bar under cyclic loading in concrete. Therefore, this study is of use for predicting the behaviour of steel bars in concrete and for the early detection of the condition of the concrete; the steel in the concrete can be applied to maintain the performance of the whole structure.

## 4. Conclusions

The magnetic leakage flux signal results from the Hp-2 signal values show high stress concentration zones in the range of 147 mm to 189 mm, which represent the low peak value of the magnetic leakage flux signal. As the load increases, the signal pattern in the critical region takes on a convex shape, which produces a strong correlation R^2^ of 0.9725, as the steel bar in the concrete continuously deforms as the cyclic load continuously acts on the beam.

The relationship between crack opening and load cycles with an R^2^ of 0.99 for beam FT10 shows a strong correlation between crack width and load cycles. An equation between magnetic flux leakage and crack opening with an R^2^ of 0.969 was proposed.

A high acoustic emission energy and amplitude with a value of 1310 eu and 74 dB can be detected at a distance of 389 mm from the left side of the beam, as the crack occurred at this position and the MMM provided a low vortex signal.

The study is of importance for understanding the behaviour of steel bars in concrete, as it is difficult to assess the behaviour of steel bars, especially under load, by visual observation. This study is useful for predicting the fatigue life of structures subjected to cyclic loading.

## Figures and Tables

**Table 1:** Tensile strength for high strength steel bar with diameter of 10 mm.

Sample	Value
Yield load (kN)	38.08
Yield stress (N/mm^2^)	622.30
Ultimate load (kN)	44.57
Ultimate stress (N/mm^2^)	734.10
Fracture load (kN)	31.50
Elongation (mm)	10.57

**Table 2:** Cycle loading arrangements.

Samples	FT10	
P_min_ (kN)	5	
P_max_ (kN)	16	
Cycle ranges	0–100	1000–1200
	100–200	1200–1400
	200–300	1400–1600
	300–400	1600–1800
	400–500	1800–2000
	500–600	2000–2200
	600–700	2200–2400
	700–800	2400–2600
	800–900	2600–2800
	900–1000	2800–3000

## Data Availability

Not applicable.

## References

[1] Zhang X., Ou J., Wu Z. (2017). Effect of circumferentially nonuniform lateral tension on bond behavior between plain round bars and concrete: Analytical study. J. Struct. Eng..

[2] Araba A.M., Ashour A.F. (2018). Flexural performance of hybrid GFRP-steel reinforced concrete continuous beams. Compos. Part B-Eng..

[3] Wang Y., Cai G., Si Larbi A., Waldmann D., Daniel Tsavdaridis K., Ran J. (2020). Monotonic axial compressive behaviour and confinement mechanism of square CFRP-steel tube confined concrete. Eng. Struct..

[4] Prince M.J.R., Singh B. (2015). Bond strength of deformed steel bars in high-strength recycled aggregate concrete. Mater. Struct..

[5] Saleh N., Ashour A., Lam D., Sheehan T. (2019). Experimental investigation of bond behaviour of two common GFRP bar types in high-strength concrete. Constr. Build. Mater..

[6] Md Nor N., Ibrahim A., Muhamad Bunnori N., Mohd Saman H., Mat Saliah S.N., Shahidan S. (2014). Diagnostic of fatigue damage severity on reinforced concrete beam using acoustic emission Technique. Eng. Fail. Anal..

[7] Noorsuhada M.N. (2016). An overview on fatigue damage assessment of reinforced concrete structures with the aid of acoustic emission technique. Constr. Build. Mater..

[8] Saliba J., Mezhoud D. (2019). Monitoring of steel-concrete bond with the acoustic emission technique. Theor. Appl. Fract. Mech..

[9] Tsangouri E., Aggelis D.G. (2019). A review of acoustic emission as indicator of reinforcement effectiveness in concrete and cementitious composites. Constr. Build. Mater..

[10] Chai M., Hou X., Zhang Z., Duan Q. (2022). Identification and prediction of fatigue crack growth under different stress ratios using acoustic emission data. Int. J. Fatigue.

[11] Verstrynge E., Charlotte V.S., Vandecruys E., Wevers M. (2022). Steel corrosion damage monitoring in reinforced concrete structures with the acoustic emission technique: A review. Constr. Build. Mater..

[12] Feng H., Shang H., Yang J., Hu B., Zhao W. (2022). Study on the bond behavior between steel bar with different derusting methods and concrete. J. Build. Eng..

[13] Wang X., Dong S., Ashour A., Ding S., Han B. (2021). Bond behaviors between nano-engineered concrete and steel bars. Constr. Build. Mater..

[14] Gao D., Huang Y., Chen G., Yang L. (2020). Bond stress distribution analysis between steel bar and steel fiber reinforced concrete using midpoint stress interpolation method. Constr. Build. Mater..

[15] Sahadan S.N., Abdullah S., Arifin A., Singh S.S.K. (2021). Assessing the magnetic flux leakage contraction parameters for the fatigue life prediction of SAE1045 steel specimens. Structures.

[16] Zhang H., Qiu J., Xia R., Cheng C., Zhou J., Jiang H., Li Y. (2022). Corrosion damage evaluation of loaded steel strand based on self-magnetic flux leakage. J. Magn. Magn. Mateial.

[17] Firdaus S.M., Arifin A., Abdullah S., Singh S.S.K., Md Nor N. (2021). Evaluating the damage mechanism characteristics for tower crane pulley using magnetic flux polar mapping distribution. J. Fail. Anal. Prev..

[18] Ege Y., Coramik M. (2018). A new measurement system using magnetic flux leakage method in pipeline inspection. Measurement.

[19] Karthik M.M., Terzioglu T., Hurlebaus S., Hueste M.B., Weischedeld H., Stamm R. (2019). Magnetic flux leakage technique to detect loss in metallic area in external post-tensioning systems. Eng. Struct..

[20] Xie Z., Zhang D., Ueda T., Jin W. (2022). Fatigue damage analysis of prefabricated concrete composite beams based on metal magnetic memory technique. J. Magn. Magn. Mater..

[21] Gong Y., Zhou J., Zhao R., Qu Y., Tong K. (2022). Study on stress measurement for steel bars inside RC beams based on self-magnetic flux leakage effect. J. Magn. Magn. Mater..

[22] Md Nor N., Abdullah S., Mat Saliah S.N. (2021). On the need to determine the acoustic emission trend for reinforced concrete beam fatigue damage. Int. J. Fatigue.

[23] Mohamad Halim M.A.H. (2022). The behaviour of steel bar embedded in reinforced concrete prisms using magnetic flux leakage, Final Year Project. Univ. Teknol. MARA.

[24] (1997). Structural Use of Concrete, Part 1: Code of Practice for Design and Construction.

[25] (2002). Standard Test Method for Flexural Strength of Concrete (Using Simple Beam with Third-Point Loading).

[26] Wang H.P., Dong L.H., Dong S.Y., Xu B.S. (2014). Fatigue damage evaluation by metal magnetic memory testing. J. Cent. South Univ..

[27] Kang C., Cui L., Zhang J., Gao L., Xu Y. (2011). Experiment research on the metal magnetic memory in gear micro crack detection. Proceedings of the IEEE International Conference on Mechatronics and Automation.

[28] Huang H., Jiang S., Liu R., Liu Z. (2014). Investigation of magnetic memory signals induced by dynamic bending load in fatigue crack propagation process of structural steel. J. Nondestruct. Eval..

[29] Shams S., Ghorbanpoor A., Lin S., Azari H. (2018). Nondestructive testing of steel corrosion in prestressed concrete structures using the magnetic flux leakage system. Transp. Res. Board.

[30] Huang H., Huang H., Jiang S., Wang Y., Zhang L., Liu Z. (2014). Characterization of spontaneous magnetic signals induced by cyclic tensile stress in crack propagation stage. J. Magn. Magn. Mater..

[31] Dong L.H., Xu B.S., Wang H.P., Xue N. (2012). A physical model for self-emitting magnetic signals during fatigue crack propagation. Appl. Mech. Mater..

[32] Liang B., Gong J.M., Wang H.T., Ye C. (2012). Evaluation of residual stresses in butt-welded joints by residual magnetic field measurements. Appl. Mech. Mater..

[33] Qiu J., Zhou J., Zhao S., Zhang H., Liao L. (2020). Statistical quantitative evaluation of bending strength of corroded RC beams via SMFL technique. Eng. Struct..

[34] Li S., Wu Y., Li W., Li P. (2021). Shear test on damage evolution of brick masonry based on acoustic emission technique. Constr. Build. Mater..

